# An intensive milk replacer feeding program benefits immune response and intestinal microbiota of lambs during weaning

**DOI:** 10.1186/s12917-018-1691-x

**Published:** 2018-11-26

**Authors:** Qian Zhang, Chong Li, Xiaolin Niu, Zhian Zhang, Fadi Li, Fei Li

**Affiliations:** 1 State Key Laboratory of Grassland Agro-ecosystems; Key Laboratory of Grassland Livestock Industry Innovation, Ministry of Agriculture and Rural Affairs; College of Pastoral Agriculture Science and Technology, Lanzhou University, Lanzhou, China; 2 College of Animal Science and Technology, Gansu Agricultural University, Lanzhou, China

**Keywords:** Lamb, Weaning stress, Immune response, Cytokine, Intestinal microbiota

## Abstract

**Background:**

Pre-weaning milk replacer (MR) feeding program is a key factor affecting the health and welfare of lambs during their weaning. Weaning stress is well known as an inducement that negatively impacts the immune system of young ruminants, whose physiological and immune state is closely linked to the community of microbiota in their intestines. This study had two objectives: 1) To evaluate the innate immune response to weaning stress at both the physiological and molecular level; 2) To investigate changes to the jejunal chyme and mucosal adhesive microbiota between the control and high plane of MR groups.

**Results:**

In this experiment, the plasma concentrations of cortisol, norepinephrine (NE) and tumor necrosis factor-α (TNFα) were higher in the C than the H group (*P* < 0.05), as was the expression of pro-inflammatory cytokines such as *TNFα* and *CXCL8* (P < 0.05) in plasma. In jejunal tissue, the expression of *TLR4* and *TNFα* were also higher in the C group (*P* < 0.01); histopathology showed the H group had lower lymphocyte infiltration. In the C group, however, major pathological changes were associated with extensive infiltration of lymphocytes, eosinophils, and neutrophils. Principal component analysis indicated the lamb immune response was influenced by weaning stress and modulated by the MR treatments. 16S-rRNA sequencing was used to evaluate jejunal mucosa and chyme bacterial diversity and composition. The C group’s chyme had a greater alpha index (ACE: *P* = 0.095; Chao1: *P* = 0.085) than H group. In jejunal mucosa, the relative abundance of *Plesiomonas* was 4-fold higher (*P* = 0.017) in the C than the H group.

**Conclusions:**

This study’s results revealed that weaning stress induced alterations to the lambs’ immune system that lasted beyond the 21 d measured, and that a long-term inflammatory response effect was evidenced by changes in their hematological and expressed pro-inflammatory cytokines. Pre-weaning with a differing MR allowance resulted in complicated biological responses and compositional changes to the lambs’ jejunal microbiota. Clearly, an intensive MR feeding program induced a milder immunity response and lower relative abundance of pathogenic bacteria when compared with the traditional feeding program.

## Background

The traditional sheep industry system has been transformed into an intensive one in China, and artificial milk replacer feeding programs have been recommended instead of ewe milk to feed the lambs, which can help to shorten the lambing interval for the ewes. However, lambs are born as pseudo-monogastrics—without a functional digestive system and immune systems—and weaning stress adversely impacts the immune system of young ruminants [1–5]. Such alterations in immunity are thought to be associated with animal growth, welfare, and disease susceptibility [6–8].

Immune responses induced by weaning stress are typically assessed in animals by considering variation in their hematological profiles [4, 5, 9]. Typically, this includes alterations of norepinephrine and cortisol levels [3, 9–11], increases of the plasma acute phase proteins concentrations [3, 9], up-regulation of gene expression involved in the pro-inflammatory response [4, 12], and transcriptional changes in cytokines have been reported in calves [2, 13]. Elevated concentrations of acute phase proteins and cytokines in plasma triggered by inflammatory signals and infection are thought to be an exclusive biomarker in weaned calves [2, 5, 14]. In particular, pro-inflammatory cytokines, namely interleukin-1 (IL-1), interferon-γ (IFN-γ) and TNF-α, were identified as the mediators of immunological and pathological responses to stress and infection [3, 4, 12, 15]. A general understanding of physiological stress is that short-term stress has beneficial consequences for the immune system, by improving its response to pathogenic infection [16]. By contrast, chronic stress suppresses and disrupts the immune system, thus increasing the incidence and severity of disease, with resulting modifications to immunopathology [16–18]. Ballou [6] had observed that with a higher plane of MR nutrition, Jersey calves increased their post-weaned maximal oxidative burst and whole-blood killing responses; this suggested that elevating MR nutrition may improve post-weaned resistance to disease. However, Johnston et al. [12] reported a negligible effect of nutrition on the immune response in gradually weaned beef calves. Therefore, the characteristic immune response to weaning stress remains unclear, given the immune system’s complexity and other contributing physiological factors, such as specific MR feeding programs and the weaning age [2, 4, 13, 19].

Early-life development and transformation of intestinal microbiota lays a foundation for its prolonged influence on host health [20, 21]. The accumulating evidence from studies on humans indicates that intestinal microbiota plays a key role in host health, as suggested by the close association of gut microbiota with the incidence of diabetes and colon cancer [22, 23]. Moreover, host physiology and diet nutrition directly impact the initial acquisition, development, and eventual stability of intestinal microbial ecosystems [24, 25]. Especially in young ruminants, for which colostrum and milk are shunted from the rumen to the abomasum through the esophageal groove, nutriment is primarily absorbed in the small intestine [26, 27]. Therefore, different MR feeding programs may affect the physiological state of the host animal by altering its intestinal microbiota, thus causing differential responses to weaning stress. In the conventional understanding of pre-weaning milk feeding programs, high levels of MR are thought increase average daily gain of body weight before weaning, yet they also reduce starter consumption and cause lower nutrient digestibility and lead to eventual body weight loss after weaning [28–30]. To date, however, there is surprisingly little information regarding the weaning stress in young ruminants and the effects of intensive feeding programs on their intestinal microbiota. Identifying intestinal microbiota changes in different planes of MR feeding lambs would be useful for understanding the internal relationship between their immune response to weaning stress and a pre-weaning milk feeding program.

Here, we hypothesized that an intensive MR feeding program could influence the hosts’ physiological state by changing not only its nutritional conditions but also its intestinal microbiota, thus inducing different immune responses to weaning stress. Therefore, the objectives of the present study were two-fold: 1) To evaluate the innate immune response to weaning stress at the physiological and molecular levels when feeding lambs normal or a high plane of MR; 2) To investigate the jejunal chyme and mucosal tissue for their adhesive microbiota alterations between the control and high plane of MR groups of experimental lambs.

## Results

### Hormone, haptoglobin and TNFα response

There were significant effects of MR treatment on NE and HP, and the treatment × sampling time interaction on NE concentration (Table 1). At 1 d, plasma cortisol concentration increased by 23% in the C group compared with 0 d, and the level was significantly higher than that in the H group. At 2 d, the plasma NE concentration increased in the C group, this exceeded that of the H group at 1 d and 2 d. After weaning, the plasma HP concentration significant decreased at 1 d, 3 d and 7 d in the H group, but there was no significant change in the C group over time. The concentration of TNFα in plasma increased by 19% at 1 d compared with the pre-weaning baseline in the C group, and it was greater than that of H group at 1 d.

**Table 1:** Effect of weaning stress on circulating plasma cortisol, norepinephrine, haptoglobin, and tumor necrosis factor concentrations

Variable		Days post weaning^1^						*P*-values^3^		
	Group	0	1	2	3	7	SEM	T^2^	S	T × S
Cortisol (ng/mL)	C	115.77	142.64^a,x^	120.33	119.62	120.12	2.40	NS	NS	NS
	H	122.53	111.47^y^	118.85	112.49	111.87	2.11			
NE (ng/mL)	C	1412.86	1543.44^x^	1668.37^b,x^	1515.79	1431.62	29.91	*	NS	*
	H	1472.32	1337.93^y^	1335.44^y^	1340.49	1363.97	31.75			
HP (ng/mL)	C	51.34	52.72^x^	52.40	50.42^x^	50.24^x^	1.33	**	NS	NS
	H	53.46	45.16^a,y^	46.87	42.81^b,y^	40.67^c,y^	1.48			
TNFα (ng/mL)	C	91.86	109.35^a,x^	100.13	94.80	94.78	1.74	NS	NS	NS
	H	98.21	88.66^y^	97.17	88.20	96.53	2.56			

Values are expressed as least squares means (LS-means)

^1^1, 2, 3, 7 d relative to weaning (= 0 d)

^2^T: MR treatment; S: sampling time; T × S: MR treatment × sampling time interaction

^3^Superscripts a, b, c within rows to indicate the LS-means differ from 1, 2, 3, and 7 d compared with 0 d by *P* < 0.05, *P* < 0.01, and *P* < 0.001, respectively; x, y within columns, to indicate that the LS-means differ between the C and H groups by *P* < 0.05; * *P* < 0.05; ** *P* < 0.01, NS: not significant (*P* > 0.05)

### Hematological responses

There were significant effects of treatment on RBC and HGB, of sampling time on total leukocyte count, lymphocyte numbers, neutrophil numbers, RBC, and HGB, and of treatment × sampling time interaction on neutrophils, RBC and HGB (Table 2). After weaning the lambs, those in C group had their total leukocyte number increased by 24% at 1 d and 14% at 2 d relative to the baseline, while those in the H group increased at 1 d only. Lymphocyte numbers significantly increased at 7 d and 14 d in the C group and at 7 d in the H group. In the C group, neutrophil count significantly increased by 40% at 1 d, and 26% at 2 d, whereas this only increased at 3 d in the H group. Neutrophils were significantly higher in the C than the H group at 1 d, and 2 d, and the former had a greater N:L ratio at 2 d. The RBC count was greater in the H group than the C group from 2 d to 21 d, significantly increasing in the former from 1 d to 21 d after weaning. The plasma HGB concentration also increased in the H group, at 1 d, 2 d, 3 d and 21 d relative to baseline, resulting in significantly higher levels when compared with the C group during the experiment.

**Table 2:** Effect of weaning stress on leukocytes, red blood cell number (RBC), neutrophil: lymphocyte ratio, and hemoglobin concentration (HGB)

Variable		Days post weaning^1^								*P*-values^3^		
	Group	0	1	2	3	7	14	21	SEM	T^2^	S	T × S
Total leukocytes (× 10^9^cells/L)	C	8.56	10.58^c^	9.76^a^	9.28	9.39	9.18	8.61	0.18	NS	**	NS
	H	8.42	9.52^a^	8.94	9.09	9.25	9.07	8.90	0.14			
Lymphocytes (×10^9^ cells/L)	C	3.44	4.12	3.68	3.95	4.44^a^	4.50^a^	3.80	0.13	NS	*	NS
	H	3.57	3.87	4.21	3.97	4.58^a^	4.08	3.66	0.12			
Neutrophils (×10^9^ cells/L)	C	3.68	5.14^c,x^	4.62^a,x^	4.06	4.37	3.69	3.26	0.14	NS	**	0.026
	H	3.39	3.97^y^	3.71^y^	4.21^a^	3.72	3.96	3.81	0.10			
N:L ratio	C	1.07	1.32	1.31^x^	1.04	1.00	0.83	0.90	0.05	NS	NS	NS
	H	0.99	1.06	0.94^y^	1.14	0.86	1.01	1.17	0.04			
RBC (× 10^12^ cells/L)	C	8.20	8.27	7.96^x^	7.93^x^	7.95^x^	8.20^x^	8.49	0.07	***	***	***
	H	8.09	8.66^b^	8.85^c,y^	8.85^c,y^	8.55^a,y^	8.66^b,y^	9.81^c,y^	0.08			
HGB (g/L)	C	114.09	115.96^x^	108.59^x^	107.96^x^	103.96^bx^	111.09^x^	120.34^x^	1.44	***	***	**
	H	116.29	124.54^a,y^	126.91^b,y^	127.16^b,y^	120.79^y^	122.29^y^	138.16^c,y^	1.13			

Values are expressed as least squares means (LS-means)

^1^1, 2, 3, 7, 14, and 21 d relative to weaning (= 0 d)

^2^T: MR treatment; S: sampling time; T × S: MR treatment × sampling time interaction

^3^Superscripts a, b, c within rows to indicate the LS-means differ from 1, 2, 3, 7, 14, and 21 d compared with 0 d by *P* < 0.05, *P* < 0.01, and *P* < 0.001, respectively; x, y within columns, to indicate that the LS-means differ between the C and H groups by *P* < 0.05; * *P* < 0.05, ** *P* < 0.01, *** *P* < 0.001, NS: not significant (*P* > 0.05)

### Whole blood cytokines and immunological biomarkers genes expression

There was a significant effect of treatment on *CXCL8*, *IL-1β*, *GRα*, *TLR4*, *TNFα*, *IFN-γ*, *NFκB2*, and *CD62L*, and of sampling time on *CXCL8*, *IL-1β*, *GRα*, *TLR4*, *TNFα*, Fas, *IFN-γ*, *NFκB1*, *NFκB2*, and *CD62L*. A treatment × sampling time interaction was detected for *CXCL8*, *IL-1β*, *GRα*, *TLR4*, *TNFα*, *NFκB2*, and *CD62L* (Table 3 and Table 4). In the C group, the expression of *CXCL8* increased from 1 d to 21 d since weaning, but there was no significant change in the H group. In addition, the C group had greater expression of *CXCL8* versus the H group from 3 d to 21 d, while the expression of *IL-1β* significantly increased in both C and H groups, though it was higher in latter from 1 d to 7 d. The expression of *GRα* increased at 1 d and did not return to pre-weaning levels in either group, yet the C group had a greater expression of *GRα* than did the H group from 3 d to 14 d. The expression of *TLR4* significantly increased in the C and H groups at 7, 14, 21 d and 1, 2, 3 d, respectively. The expression of *TNFα* significantly increased at 2 d and 14 d in the H group. In the C group, the expression of *TNFα* had a significant increase from 1 d to 7 d, and this was higher than that of the H group at 2 d, 3 d, and 7 d. The expression of *Fas* increased at 14 d in the C group, as did the *IFN-γ* expression at 1 d, 3 d, and 7 d when it also was significant greater than that of the H group. The expression of *NFκB1* significantly decreased at 2 d, but then increased from 3 d to 21 d in the C group; similarly, it significantly decreased at 2 d in the H group but then increased at 3 d, 14 d, and 21 d. The expression of *NFκB2* decreased at 3 d in the H group, but it was significantly increased at 21 d in both groups. The expression of *CD62L* significantly increased in the C and H groups on 1, 2, 7, and 14 d and 1, 14, and 21 d, respectively.

**Table 3:** Effect of weaning stress on the relative gene expression of CXCL8, IL-1β, GRα, TLR4, TNFα

Variable	Days post weaning^1^									*P*-values^3^		
	Group	0	1	2	3	7	14	21	SEM	T^2^	S	T × S
CXCL8	C	1.65	3.16^c^	3.27^c^	4.34^c,x^	4.11^c,x^	4.13^c,x^	3.52^c,x^	0.15	**	***	***
	H	2.46	2.69	2.97	2.18^y^	2.09^y^	2.41^y^	2.34^y^	0.12			
IL-1β	C	1.89	2.09^x^	1.77^x^	3.22^c,x^	3.17^b,x^	4.22^c^	5.40^c,x^	0.20	**	***	***
	H	2.68	3.37^y^	4.11^c,y^	5.37^c,y^	4.70^c,y^	4.77^c^	4.04^c,y^	0.16			
GRα	C	1.53	2.72^c^	4.92^c^	5.94^c,x^	6.97^c,x^	6.70^c,x^	3.63^c^	0.28	***	***	***
	H	1.06	2.07^b^	4.50^c^	4.30^c,y^	3.17^c,y^	3.57^c,y^	3.58^c^	0.18			
TLR4	C	2.55	2.36^x^	2.94^x^	3.02^x^	3.63^b^	4.43^c,x^	3.84^b^	0.14	***	***	***
	H	3.00	4.04^b,y^	5.50^c,y^	4.48^c,y^	3.06	2.60^y^	3.12	0.16			
TNFα	C	2.63	3.80^c^	7.29^c,x^	7.96^c,x^	6.74^c,x^	2.79^x^	2.90	0.32	***	***	***
	H	2.42	3.22	3.69^b,y^	2.96^y^	2.57^y^	3.73^b,y^	2.39	0.13			

Values are expressed as least squares means (LS-means)

^1^1, 2, 3, 7, 14, and 21 d relative to weaning (= 0 d)

^2^T: MR treatment; S: sampling time; T × S: MR treatment × sampling time interaction

^3^Superscripts a, b, c within rows to indicate the LS-means differ from 1, 2, 3, 7, 14, and 21 d compared with 0 d by *P* < 0.05, *P* < 0.01, and *P* < 0.001, respectively; x, y within columns, to indicate that the LS-means differ from C and H groups by P < 0.05; * *P* < 0.05, ** *P* < 0.01, *** *P* < 0.001

**Table 4:** Effect of weaning stress on the relative gene expression of Fas, IFN-γ, NFκB1, NFκB2, CD62L

Variable	Days post weaning^1^									*P*-values^3^		
	Group	0	1	2	3	7	14	21	SEM	T^2^	S	T × S
Fas	C	3.52	3.13	3.13	3.00	3.65	4.51^a^	3.42	0.14	NS	**	NS
	H	3.14	3.32	2.65	3.34	3.73	4.07	3.37	0.15			
IFN-γ	C	1.85	2.60^a,x^	2.48	2.58^a,x^	3.20^c,x^	2.38	1.73	0.12	**	*	NS
	H	1.36	1.74^y^	2.04	1.44^y^	1.82^y^	1.81	1.71	0.10			
NFκB1	C	2.68	2.69	1.83^a^	3.55^a^	3.65^a^	4.82^c^	4.16^c^	0.16	NS	***	NS
	H	2.82	3.11	1.24^c^	3.74^a^	2.94	4.54^c^	3.81^a^	0.19			
NFκB2	C	2.54	2.06	2.16	2.47^x^	2.30	2.63	4.54^a^	0.15	*	***	*
	H	2.36	2.15	1.93	1.09^c,y^	2.23	2.54	3.99^c^	0.13			
CD62L	C	1.86	4.01^c^	3.34^c,x^	2.41^x^	3.00^a^	3.05^b^	2.48	0.14	*	***	**
	H	1.80	3.17^b^	1.80^y^	1.35^y^	2.25	3.17^b^	3.27^c^	0.16			

Values are expressed as least squares means (LS-means)

^1^1, 2, 3, 7, 14, and 21 d relative to weaning (= 0 d)

^2^T: MR treatment; S: sampling time; T × S: MR treatment × sampling time interaction

^3^Superscripts a, b, c within rows to indicate the LS-means differ from 1, 2, 3, 7, 14, and 21 d compared with 0 d by *P* < 0.05, *P* < 0.01, and *P* < 0.001, respectively; x, y within columns, to indicate that the LS-means differ from C and H groups by P < 0.05; * *P* < 0.05, ** *P* < 0.01, *** *P* < 0.001, NS: not significant (*P* > 0.05)

### Principal component analysis

**Fig. 1:**
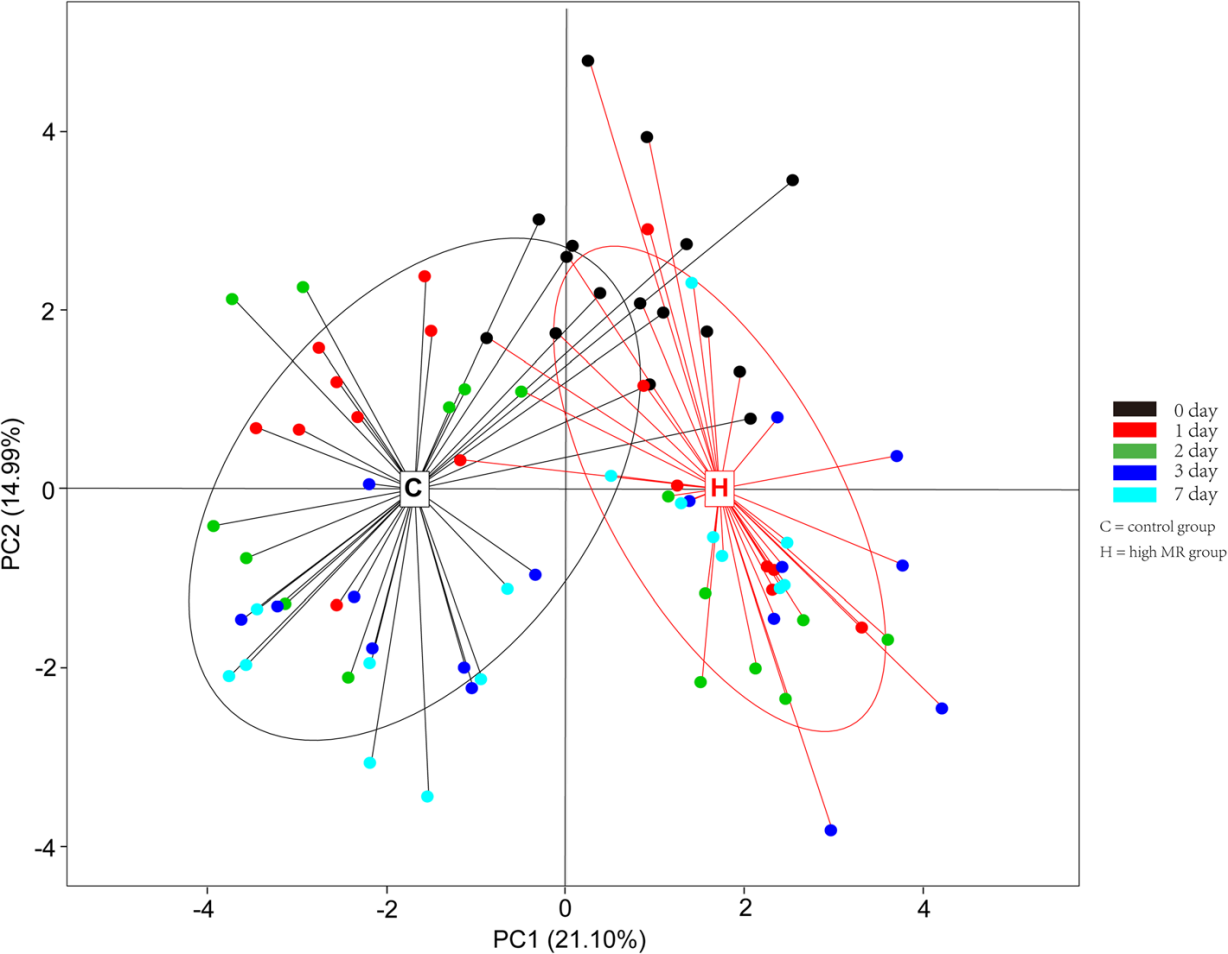
Principal component analysis (PCA) of blood indices for the different groups

The hematology, acute phase protein, norepinephrine, cortisol, and whole blood cytokines genes’ expression were evaluated by a PCA, which reduced the dimensions of the original variables (Fig. 1). This analysis indicated that the control vs. high group MR treatment of lambs separated into two clusters on 1, 2, 3, and 7 d after weaning.

### Jejunal mucosa and chyme microbiota diversity and community structure

**Fig. 2:**
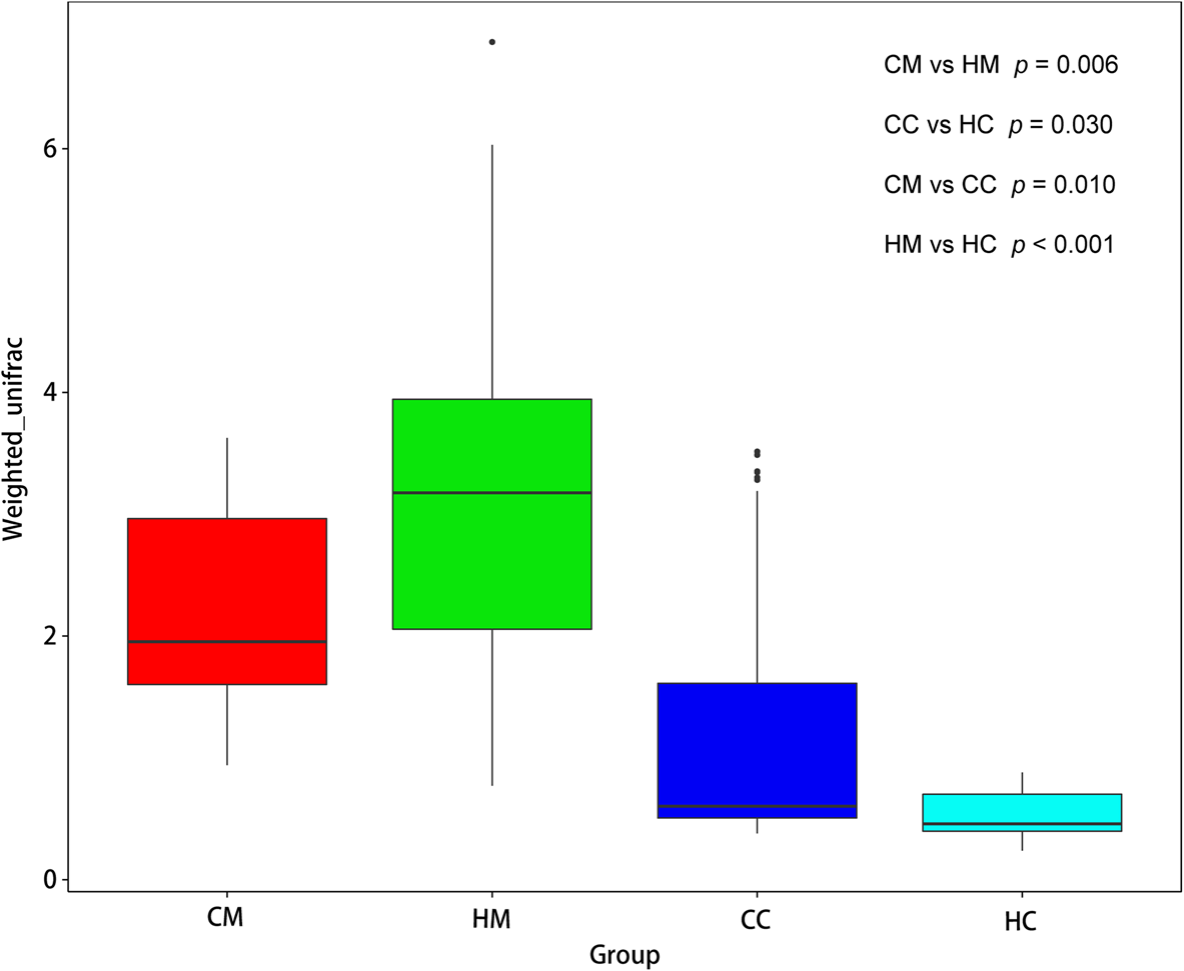
Boxplots of weighted UniFrac beta diversity among different groups by Tukey test. CM: control group, mucosa; HM: high plane group, mucosa; CC: control group, chyme; HC = high plane group, chyme

**Fig. 3:**
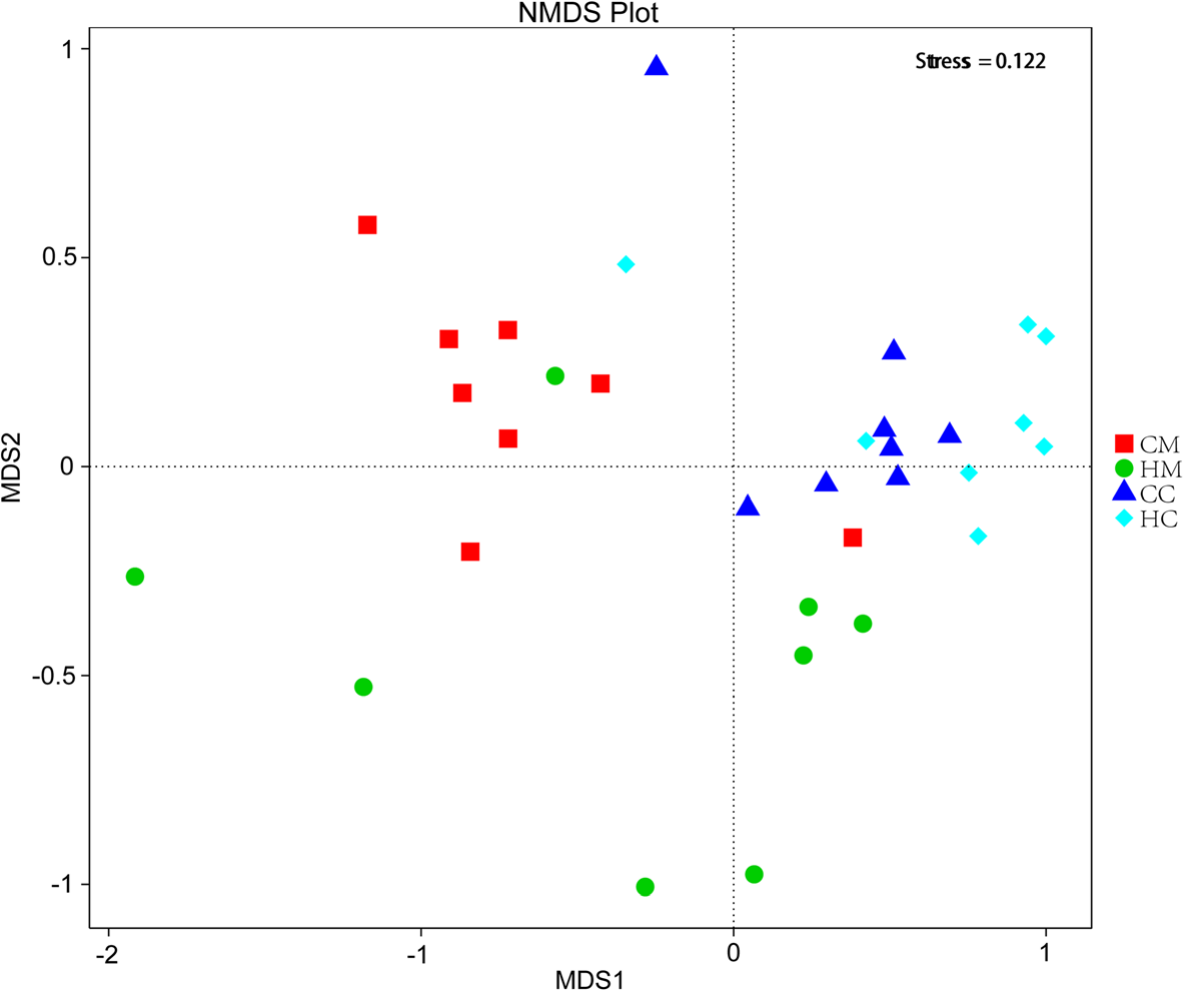
Non-metric multi-dimensional scaling (NMDS) analysis of the jejunal bacterial OTUs for the different groups. CM: control group, mucosa; HM: high plane group, mucosa; CC: control group, chyme; HC: high plane group, chyme

**Fig. 4:**
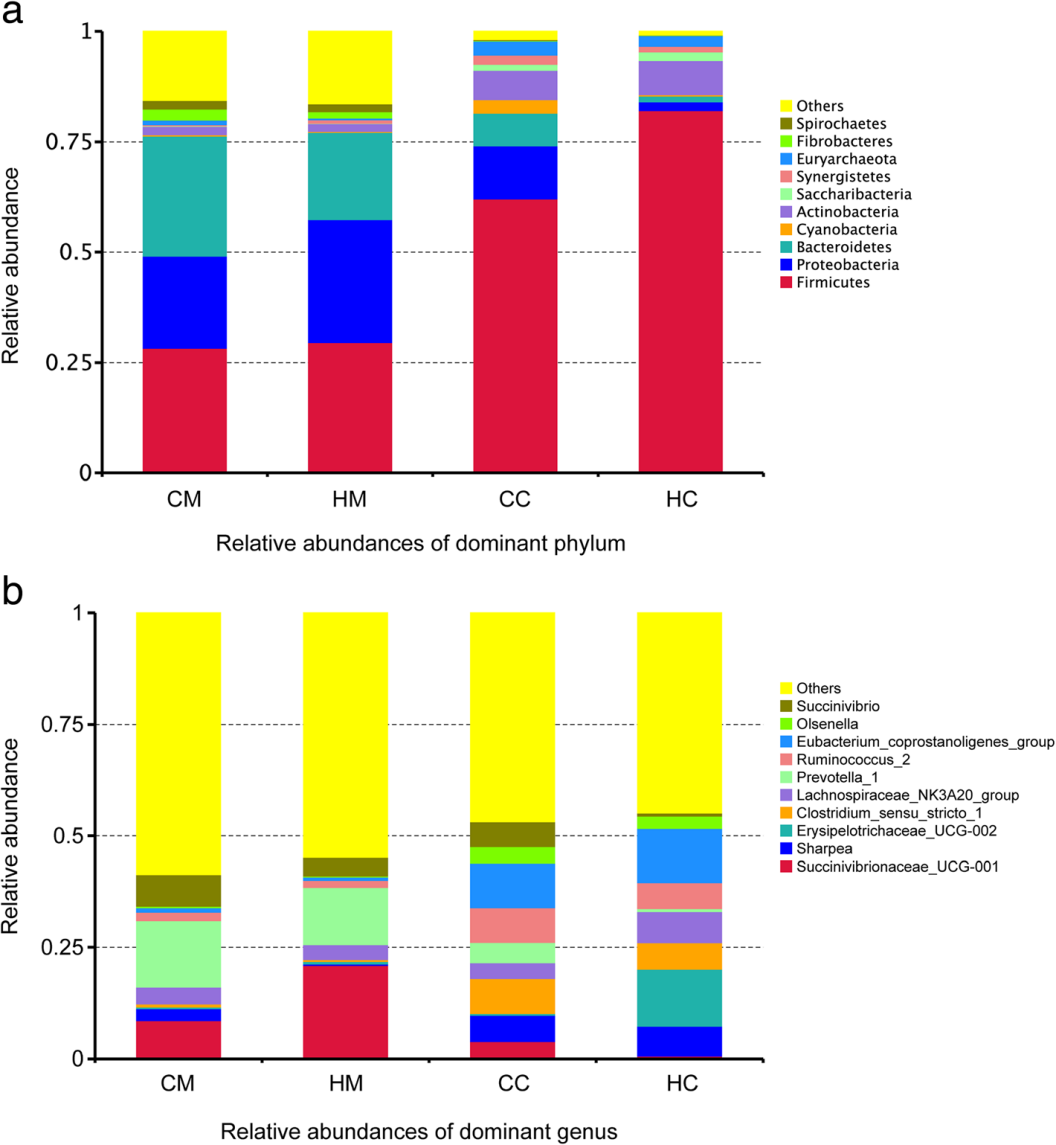
Top 10 most abundant dominant phyla (**a**) and genera (**b**) in jejunal mucosa and chyme. CM: control group, mucosa; HM: high plane group, mucosa; CC: control group, chyme; HC: high plane group, chyme

Alpha diversity analysis results are listed in Table 5. The C group showed higher OTU, ACE, Chao1, Shannon, Simpson index values for mucosa and chyme, and had a trend (*P* < 0.10) of greater ACE index, Chao1 index and Shannon index in chyme. The library coverage of samples from the two groups was above 99%. The OTU β-diversity was determined by using the phylogeny-based UniFrac method. An analysis of the weighted UniFrac distances (Fig. 2) also indicated significant differences in the beta diversity community composition between the groups. The NMDS plot showing the dissimilarity of microbial community also revealed distinct structures of the groups (Fig. 3). The stress values for the four ordination plots were < 0.2 which indicates these data were well-represented by the 4-dimensional representation used. The top 10 relatively abundant phyla and genus are presented in Fig. 4. Both in mucosa and chyme, the most abundant phyla were Firmicutes in the C and H groups (relative abundances of 28.315, 29.605, 62.015, and 82.15%, respectively). In the jejunal mucosa, the most abundant genus was *Prevotella_1* (relative abundance of 14.93%) in the C group, but it was *Succinivibrionaceae_UCG-001* (relative abundances of 20.98%) in the H group. In the jejunal chyme, the most abundant genus was *Eubacterium_coprostanoligenes_group* (relative abundance of 10.02%) in the C group, but it was *Erysipelotrichaceae_UCG-002* (relative abundance of 12.80%) in the H group. The mean relative abundance of bacterial taxa present at > 0.1% in the jejunal mucosa and chyme, which were significantly different at the phylum and genus levels between C and H groups, are listed in Table 6. In jejunal mucosa, the relative abundance of Fusobacteria was higher in the C than H group. At the genus level, in jejunal mucosa the relative abundances of *Eubacterium_nodatum_group*, *Plesiomonas*, *Cetobacterium*, *Lactococcus*, *Streptococcus*, *Ruminococcaceae_UCG-004*, all were higher in the C group than the H group, whereas, *Oribacterium* was higher in the H group. In jejunal chyme, the relative abundance of Firmicutes was higher in the H group than in the C group; however the latter had a greater relative abundance of Proteobacteria than the former. At the genus level, the relative abundances of *Succinivibrio*, *Prevotella_7*, *Desulfovibrio*, and *Bacteroides* were higher in the C than the H group, yet *Erysipelotrichaceae_UCG-002* was higher in the H group.

**Table 5:** Effects of milk replacer on the alpha diversity index of 21-d weaned lambs

Diversity index	Group				Group			
	CM	HM	SEM	P-value	CC	HC	SEM	*P*-values
OTU	849.1	700.5	51.37	0.154	562.0	450.3	35.26	0.116
ACE	903.3	740.6	64.86	0.221	585.8	439.9	43.56	0.095
Chao1	886.9	735.1	62.72	0.239	571.5	423.6	43.04	0.085
Shannon	6.284	5.731	0.218	0.216	5.103	4.429	0.194	0.081
Simpson	0.953	0.914	0.014	0.185	0.910	0.880	0.011	0.203
Coverage (%)	0.992	0.993	0.001	0.307	0.995	0.996	< 0.001	0.165

*CM* Control group, mucosa, *HM* High plane group, mucosa, *CC* Control group, chyme, *HC* High plane group, chyme

**Table 6:** Relative abundances of bacterial taxa (%) in the jejunal mucosa and chyme

Items	C	H	SEM	*P*-value
*Jejunal mucosa*				
Phylum				
Fusobacteria	0.58	0.09	0.001	0.015
Genus				
Oribacterium	2.87	6.11	0.008	0.043
Eubacterium_nodatum_group	0.56	0.25	0.001	0.019
Plesiomonas	0.75	0.18	0.001	0.017
Cetobacterium	0.55	0.06	0.001	0.017
Lactococcus	0.23	0.08	< 0.001	0.043
Streptococcus	0.16	0.05	< 0.001	0.049
Ruminococcaceae_UCG-004	0.17	0.09	< 0.001	0.048
*Jejunal chyme*				
Phylum				
Firmicutes	62.09	82.15	0.038	0.005
Proteobacteria	12.07	1.98	0.024	0.041
Genus				
Erysipelotrichaceae_UCG-002	0.37	12.80	0.030	0.046
Succinivibrio	5.58	0.67	0.012	0.043
Prevotella_7	0.43	0.09	0.001	0.015
Desulfovibrio	0.15	0.06	< 0.001	0.008
Bacteroides	0.20	0.08	< 0.001	0.041

*C* Control group, *H* High plane of milk replacer group

Mean abundance of bacterial taxa present at > 0.1% in the jejunal mucosa and chyme

### Jejunal tissue histomorphology and cytokines genes expression

**Fig. 5:**
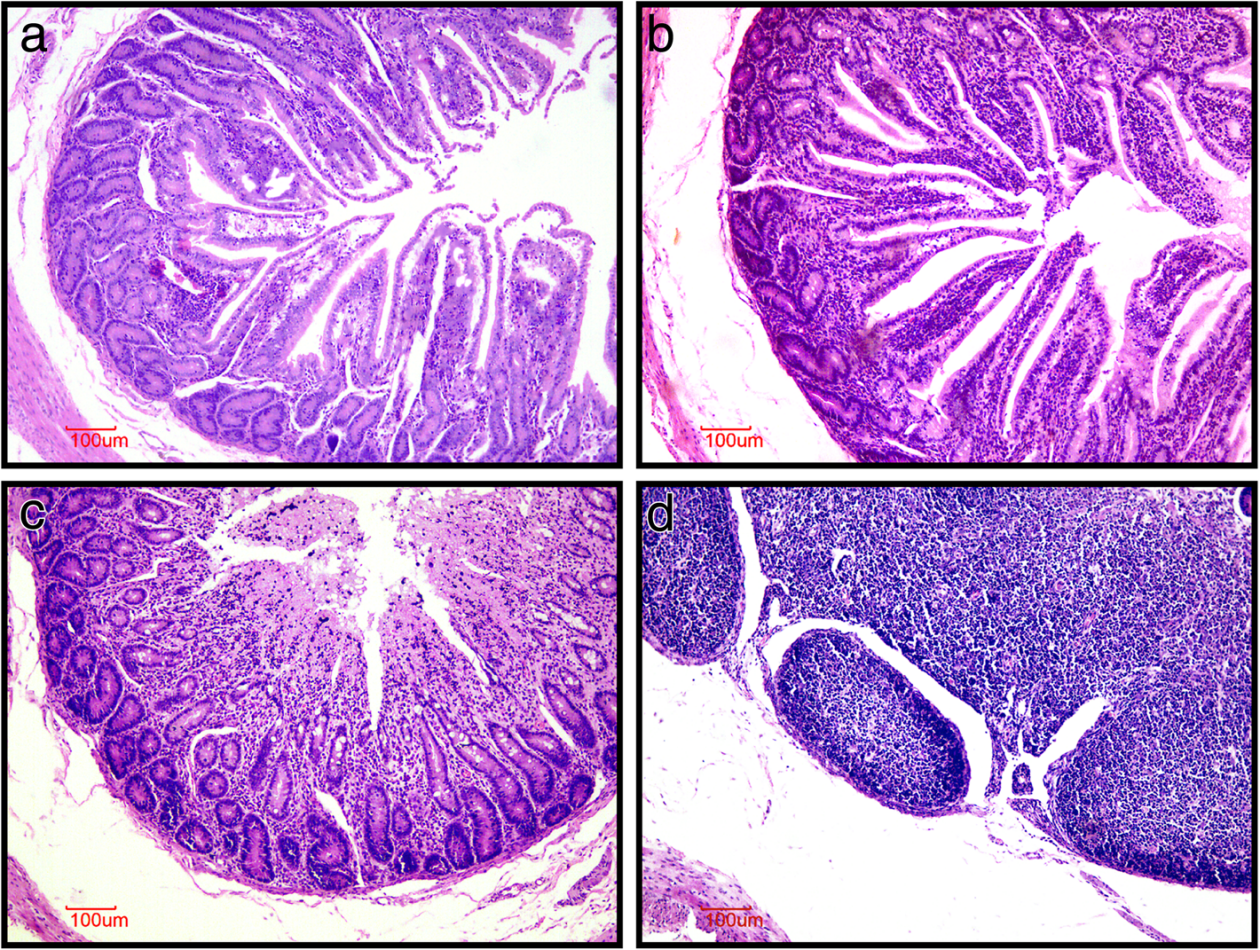
Histomorphology of the jejunal tissue of hematoxylin and eosin stained sections (× 100) of jejunal tissue. Control group (**b**, **c**, and **d**) and High plane group (**a**)

**Fig. 6:**
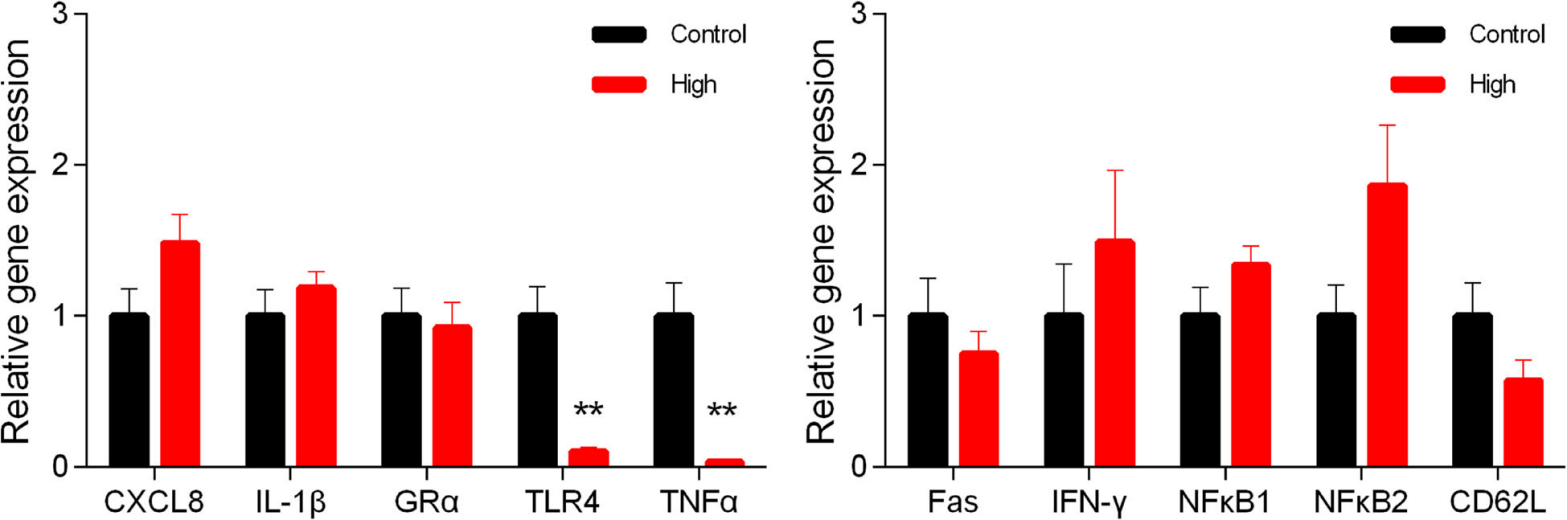
Relative gene expression (means ± SE) of jejunal tissue cytokines and immunological biomarkers. * *P* < 0.05, ** *P* < 0.01, *** *P* < 0.001

In comparison to the H group (Fig. 5a), major pathological changes in the C group were associated with an extensive infiltration of lymphocytes, eosinophils, and neutrophils into the mucosal and sub-mucosal layers (Fig. 5b). Abnormal villi were observed in many areas with an incomplete structure in the mucosal layer surrounded with lymphocytes, neutrophils, and eosinophils (Fig. 5c). Furthermore, Peyer’s patches were also frequently detected in some regions of the jejunum sub-mucosa with hyperplasia and edema (Fig. 5d) in the C group. The expression of cytokines in the jejunal mucosa was largely similar, except for *TLR4* and *TNFα*, which were more than 9-fold higher (*P* < 0.01) in the C group than H group (Fig. 6).

### Correlation analysis

**Fig. 7:**
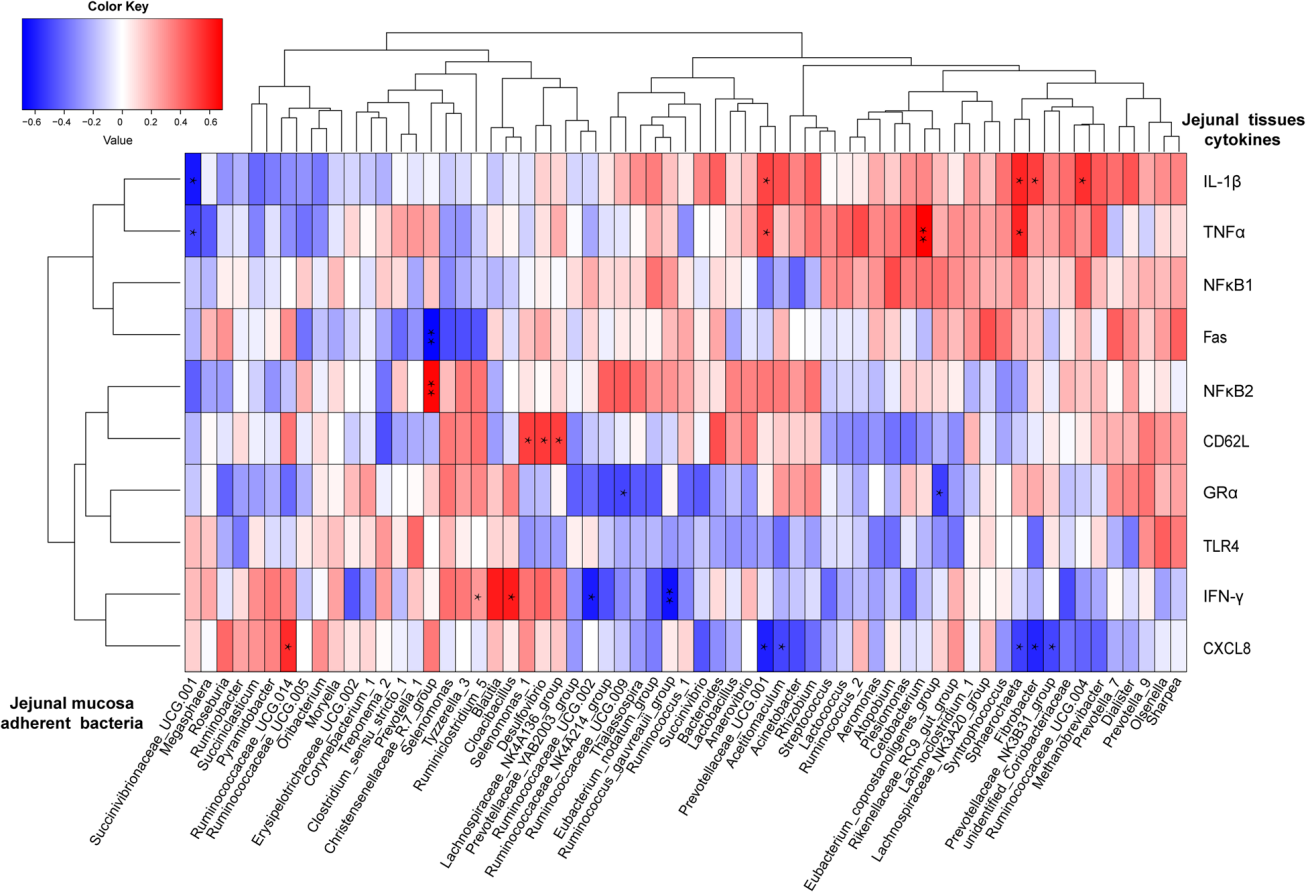
Correlations between the abundance of adherent bacterial and the gene expression of jejunal tissue cytokines. *P* < 0.05, ** *P* < 0.01, *** *P* < 0.001

Correlations of the mean abundance of bacterial taxa (present at > 0.1% of genus levels) in the jejunal mucosa-adherent bacteria with the gene expression levels of jejunal tissue cytokines based on Pearson tests are displayed in Fig. 7. *IL-1β* had a positive correlation with *Fibrobacter*, *Prevotellaceae_UCG.001*, *Sphaerochaeta* and *Ruminococcaceae_UCG.004* (*P* < 0.05) and *TNFα* had a positive correlation with *Prevotellaceae_UCG.001*, *Cetobacterium* and *Sphaerochaeta* (*P* < 0.05), yet both *IL-1β* and *TNFα* were negatively correlated with *Succinivibrionaceae_UCG.001* (P < 0.05). *Fas* was negatively correlated with *Christensenellaceae_R.7_group* (*P* = 0.005, *r* = − 0.67) but *NFκB2* was positively correlated (P = 0.005, *r* = 0.67). *CD62L* had positive correlations with *Selenomonas_1*, *Desulfovibrio* and *Lachnospiraceae_NK4A136_group* (*P* < 0.05). *GRα* had negative correlations with *Eubacterium_coprostanoligenes_group* and *Ruminococcaceae_UCG.009* (*P* < 0.05). *IFN-γ* was positively correlated with both *Cloacibacillus* and *Ruminiclostridium_5* (*P* < 0.05), yet it was negatively correlated with *Ruminococcus_gauvreauii_group* and *Ruminococcaceae_UCG.002* (*P* < 0.05). *CXCL8* was positively correlated with *Ruminococcaceae_UCG.014* (*P* = 0.020, *r* = 0.58) yet negatively correlated with *Acetitomaculum*, *Fibrobacter*, *Prevotellaceae_UCG.001*, *Sphaerochaeta* and *Prevotellaceae_NK3B31_group* (P < 0.05).

## Discussion

Weaning typically combines a number of physical and psychological stressors that have the potential to alter the animal’s immune status. The systemic reaction to a stressor encompasses a wide range of endocrinological, immunological, and inflammatory responses. An MR feeding program can influence the physiological status of the host and its immune responses to weaning stress by providing different nutritional conditions and altering its intestinal microbiota. Therefore, this study aimed to investigate the characteristics of immunological and inflammatory responses as well as of intestinal microbiota under control and high plane of MR treatments.

### Hormone and haptoglobin response

Lambs lack a fully-developed adaptive immune system, and they rely on the hypothalamo-pituitary-adrenal (HPA) axis which controls reactions to stress and serves as the primary regulator and modulator of immunity. Previous studies reported the plasma concentration of cortisol increased in calves when they were exposed to weaning stress, and can be viewed as a biomarker of stress in bovine animals [3, 9]. Furthermore, the sympatho-adrenal axis releases norepinephrine, which stimulates the immune-related cells, causing them to produce pro-inflammatory cytokines in response to stress [19, 31]. In our study, the concentrations of cortisol and NE increased relative to the baseline after weaning in the C group. This result suggests that lambs in the C group were more sensitive to weaning stress than those fed a high plane of MR; however, caution is required when drawing any conclusion, because the cortisol and NE peaks respectively occurred only at 1 d and 2 d since weaning. But this increase did not last for a long time, as found in previous studies [3, 10, 11]. This short-term change in the present study may point to a not well-developed HPA axis in the 21-d weaned lambs.

Haptoglobin has a higher sensitivity for detecting disease when compared with other acute-phase proteins, due to it is more pronounced and prolonged response to infection [32]. Previous studies have shown that weaned calves had an increased concentration of plasma HP, which was considered as a useful measure of inflammatory and stress responses in calves [5, 14, 33, 34]. Some pro-inflammatory cytokines, such as IL-1 and TNFα, could positively mediate the hepatocyte production and secretion of HP during an inflammatory response [35]. Ballou [6] reported that Holstein and Jersey calves fed the higher planes of MR nutrition had a greater HP concentration than calves fed the lower planes of MR at 24 h after the injection of lipopolysaccharide (LPS). However, we were surprised to find that the plasma HP concentration in lambs of the H group decreased after weaning, a result perhaps explained by the higher lymphocyte numbers found after weaning. The B cells dysfunction leads them to produce autoantibodies or a complement that adheres to the erythrocyte membrane, resulting in red blood cell damage and the release of hemoglobin into peripheral blood; HP then binds this hemoglobin, removing it from circulation [36]. Nevertheless, caution is necessary, because of the limited investigation into the effects of nutrition on weaning stress through either nutritional modulation or immunogenic stimulation. Further research is warranted to investigate the potential mechanism of early nutritional support to the young ruminants during the weaning phase.

### Hematological responses

We found that the different MR allowance affected the pattern of changes in other immunological and inflammatory responses after weaning. In this study, the total leukocyte number significantly changed at 2 d in the C and H groups. This increase in the weaned lambs agrees with Lynch et al. [5] and Johnston et al. [12], who observed elevated total leukocyte numbers in weaned calves with an abrupt or gradual weaning strategy, respectively. However, other studies found negligible differences in total leukocyte number between pre- and post-weaning in calves [3, 4, 9, 37]. For this reason, we speculate that blood total leukocyte number is not a reliable indicator of the immunity response in lambs after weaning. Our lambs in the C group had a 40% increase in their neutrophil number, a result consistent with the other work in which the neutrophil number increased considerably in calves after weaning [2, 4, 5, 9, 14]. Several studies suggested that an increase in the neutrophil count of circulation blood may be interpreted by a decreasing expression of *CD62L*, which could reduce the ability of neutrophil margination and subsequent migration from the vasculature [12, 38]. Yet our findings are distinct from these studies [12, 38], in that the expression of *CD62L* significantly increased in all the experimental lambs after weaning. This result indicates that the increased neutrophil numbers of weaned lambs could not have been caused by a change in an adhesion molecule on the neutrophil surface, and that weaning stress did not negatively influence neutrophils’ ability to move and adhere to the endothelium lining of blood vessels. This result is consistent with O’Loughlin et al. [4], who observed that the expression of *CD62L* increased from 1 d to 7 d in calves after weaning. Interestingly, the lymphocyte count result disagrees with other research, that has found the lymphocyte number to decrease in calves after weaning [4, 5, 39], and had attributed it to the trafficking of lymphocytes from general circulation into the tissues and organs at risk of infection [16]. In the present study, the increase of lymphocyte numbers may arise from lymphocyte dysfunction caused by the significantly increased expression of *TNFα* and *IL-1β*, which act as co-stimulators to regulate the proliferative response of thymocytes [40].

### Cytokine gene expression

We relied on several cytokines and immunological biomarkers [2–4, 12, 13] to evaluate immune response of the lambs before and after weaning. In our study, the changed TNFα was subject to a similar regulation, both in terms of its relative mRNA expression and plasma protein concentration, which significantly increased at 1 d in the C group lambs. This result is in line with O’Loughlin et al. [4], who reported the expression of *TNFα* was up-regulated in weaned calves throughout their experiment. The TNFα is a multifunctional cytokine which can induce many cellular responses, and it plays a role in the activation of an inflammatory cascade [41]. This up-regulation of TNFα closely coincided with *IFN-γ* and *IL-1β* expression. TNFα often work synergistically with IFN-γ and IL-1β to increase the margination of lymphocytes and macrophages by decreasing the local blood flow rate and causing blood to gather in leaky vessels, then leukocytes are led by cytokine and chemokine to extravasate into the inflammatory tissue [42, 43]. As such, IL-1β is a potent and potentially dangerous mediator of inflammation caused by stress [44]. In the present study, that the expression of *IL-1β* significantly increased after weaning agrees with previous research [2, 4], and it could activate T cells and promote B cell proliferation [40]. The expression of *IFN-γ* increased in the C group following an up-regulation of *TNFα*, which promotes a cell-mediated inflammatory response. This result is consistent with some other studies that suggesting IFN-γ production is stimulated by TNFα [42, 43]. Thus, the greater expression of *TNFα*, *IL-1β*, and *IFN-γ* genes most likely reflects a strongly inflammatory response and stimulation of the immune response in the C group after weaning.

CXCL8 is a crucial inflammatory mediator and neutrophil chemoattractant, and it accounts for distributional alterations in the circulating neutrophils and functions in T cell migration, serving to increase the host immune response [18, 45]. The expression of *CXCL8* was reportedly up-regulated following weaning [3, 4], yet these studies were limited to a short period of time. In our study, the expression of *CXCL8* increased at 1 d and did not return to the baseline at 21 d in the C group. Hence, this reveals a long-term effect of the inflammatory response in the C group lambs. Prolonged exposure to the CXCL8 cytokine can result deleterious effects that increase disease susceptibility and producing severe tissue damage [46]. The expression of *GRα* significantly increased at 1 d and remained so at 21 d for both treatment groups in this current study. This result suggests the adjustment period to weaning induced stress may be longer than the anticipated 21 d, an interpretation supported by O’Loughlin et al. [4] who observed the expression of *GRα* increased more than 3-fold in calves after weaning throughout their experiment. The lambs in our C group showed a higher expression level of *GRα* than the H group from 3 d to 14 d, indicative of a stronger stress-induced inflammatory dysregulation. By contrast, Johnston et al. [12] found no notable differences in *GRα* expression since weaning, perhaps because the gradual weaning strategy in that study gave the calves ample time to adapt to the weaning stress, making it less stressful overall. TLR4 has been investigated as a modulator of both innate and adaptive immunity and it plays a role in the non-infectious inflammatory response [47]. The up-regulated expression of *TLR4* in our study was similar to the results of O’Loughlin et al. [4], who found its expression was increased in calves at 1 d without a return to pre-weaning levels at 7 d. Those authors also suggested using TLR4 as a new potential biomarker of weaning stress in bovines. However, in our study, the MR treatments interacted with sampling time upon TLR4 to induce a different response between the two groups of lambs. Thus, TLR4 may in fact be an unreliable indicator of the weaning stress response, since it depends on the weaning strategy as well as the pre-weaning feeding program.

When the results of the endocrinological, immunological and inflammatory responses are considered with those of the cytokines and immunological biomarkers gene expression, it is clear that the immune response was influenced by weaning stress yet modulated by nutritional status. The PCA indicated that the MR treatments given to lambs separated into two clusters at 1, 2, 3, and 7 d after weaning. These results indicated that the inflammatory response and stimulation of the immune response differed between the C and H groups; hence these disparate responses between the treatments must be due to their different nutritional status. Protein, fatty acid, vitamin or microelement levels in a diet can affect the membrane composition of immune cells and the inflammatory response [48]. Although there have been studies focusing on the feeding regimes in their early life for the ruminants, only a few have attempted to integrate nutritional factors with immune responses induced by weaning stress. Although our study indicated that the differences in immune responses under the two MR feeding programs are mainly due to the variation of nutrient intake, there is still a need to improve our knowledge about nutritional factors that can regulate the immune response of young ruminants.

### Jejunal histomorphology, cytokines genes expression, and microbiota

We further analyzed the effect of the MR feeding programs on the jejunal histomorphology and gene expression related to intestinal innate immunity. In our study, the histopathology of the C and H group lambs reflected differential characteristics after weaning. The jejunal mucosa of the C group showed greater pathological changes, which is consistent with the jejunal mucosal inflammatory cytokines expression that was found to be higher in the C than H group. Greater *TNFα* and *TLR4* expression in the C group lambs likely resulted from lymphoid infiltration and inflammation in their jejunal mucosa. The intestinal microbiota is closely linked to the physiological status of the host, especially its immune function [25, 27], and it can directly impact the dynamic equilibrium of intestinal microbiota [24, 26]. We found that each MR feeding group sustained its own distinct microbial community, as inferred from the weighted UniFrac beta diversity and the clustering of samples by group in the NMDS. At both the phyla and genus levels, the composition and relative abundance of resident microbiota differed greatly between the jejunal mucosa and chyme. This result suggests that using the gut chyme or feces to investigate the interaction between bacteria and host is neither reliable nor accurate.

According to one study, several alpha diversity indices (Chao, ACE, and Shannon indices) were higher in the C group in chyme (*P* < 0.1), which also suggests that the microbiota were more diverse in the C group lambs. In theory, the gut bacteria diversity increases with age, and a highly diverse gut microbiota is regarded as a sign of a mature gut microbiota [24, 49]. However, some studies indicate microbiota development and diversification should not occur too earlier and quickly, since the bacteria need to develop in a gradual process; prematurely occurring changes towards an adult-type microbiota may cause damage to host gut immune function [50, 51]. This may arise because the lambs-type microbiota supports a gut barrier function as well as tolerance against allergens in an immature gut, which affects the maturation of the gut epithelium and later immune functioning. Thus, the high plane of MR may have provided indirect protection of gut immunity in lambs.

The *Clostridium* genus has been shown to be associated with inflammatory bowel disease [52, 53]. Among the lambs’ jejunal mucosa adherent microbiota, 28.57% of the different sequences found between the C and H groups came from the order *Clostridiales*, with two-thirds of them significantly more abundant in the C group and of which all belonged to *Clostridium*; i.e. *Clostridium_sensu_stricto_1*, *Clostridium_sensu_stricto_3*, *Clostridium_sensu_stricto_5*, *Clostridium_sensu_stricto_10*, *Clostridium_sensu_stricto_12*, *Clostridium_sensu_stricto_13*, and *Clostridium_sensu_stricto_15* were (collectively) more relatively abundant in the C group than H group (0.70 vs. 0.45%, *P* = 0.144). This result indicates the H group may have harbored a less relative abundance of pathogenic bacteria. At the genus level, the relative abundance of *Plesiomonas* was 4-fold higher in the C than H group in the lambs’ jejunal mucosa. *Plesiomonas* is classified in the family *Enterobacteriaceae* and has wide range of hosts, including humans and cows. In humans, *Plesiomonas* was reportedly implicated in gastrointestinal infection and diarrhea [54, 55]. The higher relative abundance of *Plesiomonas* in the C group may indicate that these lambs have a higher risk of gastrointestinal disease. However, *Plesiomonas* was uncorrelated with the gene expression of any jejunal tissue cytokines and immunological biomarkers. A plausible explanation for this is that all 16 lambs used in this experiment were healthy and without any clinical symptoms of diarrhea. Furthermore, we found several genera of bacteria that were significantly correlated to the gene expression of jejunal tissue inflammatory cytokines; this provides some useful information for studying the intestinal health of young ruminants. However, the function of these bacteria and the symbiotic relationship between intestinal microbiota and their hosts is quite complicated, hence more research is needed to better understand how intestinal microbiota and host immune function interact.

## Conclusions

The results of this study revealed that weaning stress induced alterations to the lambs’ immune system, which lasted beyond the 21 d measured, and that a long-term inflammatory response effect was evidenced by changes in their hematological and expressed pro-inflammatory cytokines. Changing the pre-weaning MR allowance resulted in complicated biological responses and compositional changes to lambs’ jejunal microbiota. It is clear that the intensive MR feeding program induced a milder immunity response and lower relative abundance of pathogenic bacteria in lambs in comparison with their traditional feeding program.

### Materials and methods

This study followed the recommendations of the Biological Studies Animal Care and Use Committee of Gansu Province, China (2005–12). The experiment was approved by Lanzhou University (2017YFD0500502), and it was conducted according to their established guidelines. All efforts were made to minimize animal suffering.

### Animal management

Twenty male Hu lambs (ordered from Minqin Zhongtian Sheep Industry Co. Ltd., Minqin, China), of the same age and similar birth weight (mean ± SE: 3.29 ± 0.13 kg), were housed indoors with their ewes from birth to 6 d, which ensured they had fed enough colostrum. At 4 d, the lambs were trained to use the nipple bottle to feed them an MR. At 7 d, the lambs were separated from their ewes and placed in a warm and ventilated nursery within individual pens (0.65-m wide × 1.10-m long; area = 0.715 m^2^). At 7 d, four lambs were removed from this study because of serious diarrhea and drug therapy. The remaining 16 healthy lambs were randomly divided into two groups (*n* = 8) that received a differing MR allowance: control (C) vs. high (H) plane. The control group received a traditional MR feeding quantity, which was 2% of average body weight (average body weight at 7 d: 4.56 kg; at 14 d: 4.97 kg) per day, following the feeding guidelines of the Feed Research Institute Chinese Academy of Agricultural Sciences, China [56]. The high group received an intensive MR feeding quantity, which was 4% of average body weight (average body weight at 7 d: 4.54 kg; at 14 d: 5.52 kg) per day. All lambs were fed the MR by a nipple bottle three times daily (at 09:00, 15:00, 21:00 h). The whole milk replacer contained 23.22% crude protein (CP) and 13.20% fat (made by the same Feed Research Institute). All lambs were weaned at 21 d.

The starter diet contained 20% CP and 18% neutral detergent fiber, formulated to meet the requirements of the feeding standard of meat-producing sheep [NYT816–2004], starter and water were supplied ad libitum from 7 d to 49 d. At 50 d, the slaughter took place in the slaughter facilities at the experimental station of Lanzhou University. The slaughtering procedures were carried out in accordance to the Biological Studies Animal Care and Use Committee of Gansu Province, China (2005–12). Before morning feeding, lambs were euthanized by penetrative captive bolt followed by exsanguination from the jugular vein was carried out; from each individual, its jejunal chyme was collected into 5-mL sterile tubes and jejunal tissue was sampled from a similar location. All jejunal chyme and tissue samples were immediately stored in the liquid nitrogen after collection, and then kept at − 80 °C until further analysis. After samples collection, all lambs were harmlessly treated by incinerator.

### Blood sample collection

Blood samples were collected from lambs via jugular venipuncture on − 3, 0, 1, 2, 3, 7, 14, and 21 d relative to weaning (= 0 d) before the morning feeding. On each occasion, the blood was drawn by the same experienced operator, who took < 60 s to collect samples from a lamb. Individual blood samples were collected into 5-mL Lithium Heparin (LH) tubes and 2 × 2 mL K_3_Ethylenediaminetetraacetic acid (K_3_EDTA) tubes. Once collected, the blood in the LH tubes was centrifuged at 3000×g for 15 min. Plasma was harvested and stored at − 20 °C until assayed. Blood in the K_3_EDTA tubes was stored at − 80 °C for cytokine gene expression profiling. The other K_3_EDTA tubes were transported to the laboratory at ambient temperature within 2 h of collection, and their hematology analyzed immediately.

### Hematology

The whole K_3_EDTA blood samples were examined using a hematology analyzer (PROKAN PE6800 Prokan Electronics Inc., Shenzhen, China) equipped with software for sheep blood. Total leukocyte, neutrophil, and lymphocyte numbers, red blood cell (RBC) counts, and hemoglobin (HGB) were measured. The neutrophil: lymphocyte (N: L) ratio was also calculated.

### Acute phase protein, norepinephrine, cortisol, and TNFα

All plasma samples were analyzed in triplicate. An automatic microplate reader (Thermo Scientific, Wilmington, USA) was used to measure the concentration of cortisol, norepinephrine, Haptoglobin (HP) and TNFα in plasma. This was done commercial assay kits (Abcam, Cambridge, UK) according to the manufacturer’s instructions.

### RNA extraction and cDNA synthesis

RNA was extracted from whole blood and jejunal tissue using the RNAiso Blood Kit (Takara, Kusatsu, Japan) and MiniBEST Universal RNA Extraction Kit (Takara, Kusatsu, Japan), both according to manufacturer’s instructions. A NanoDrop 2000 spectrophotometer (Thermo Scientific, Wilmington, USA) was used to quantify the RNA, and its integrity was assessed using 1% denaturing agarose gel electrophoresis. One μg of total RNA per animal was reverse transcribed into complementary DNA (cDNA) by using the RT Primer Mix and the PrimeScript™ RT reagent kit (Takara, Kusatsu, Japan) in a 20-μl reaction and then stored at − 20 °C.

### Real-time qPCR

All operations followed the MIQE guidelines [57]. Primers for the candidate genes (refer to Table 7) were designed based on known ovine sequences, obtained from the NCBI database, using Primer Premier v. 6.0 software (Premier Biosoft Interpairs, Palo Alto, USA). All primers were synthesized by Sangon Bio Inc. (Sangon, Shanghai, China). Serial dilutions of pooled cDNA samples were used to determine the amplification efficiencies, using the eq. E = − 1 + 10^(− 1/slope)^. The slope was calculated by plotting the linear curve of the cycle threshold (CT) values against the log dilutions [58]. Only those primers with PCR amplification efficiencies > 90% were used in our study. The relative amount of each studied mRNA was normalized to *β-actin* mRNA levels as a housekeeping gene. A real-time quantitative PCR detection system (Bio-Rad Laboratories Inc., Hercules, CA) was used to determine the relative level of mRNA expression. Each 25-μL real-time PCR reaction contained 2 μL of cDNA, 12.5 μL of SYBR Premix Ex Taq Perfect Real Time (Takara, Kusatsu, Japan), 0.4 μL of each forward and reverse primers, and 9.7 μL of ddH_2_O; performed under denaturization conditions with the following program: 95 °C for 30 s followed by 40 cycles of 95 °C for 5 s and 60 °C for 30 s, finishing with amplicon dissociation at 95 °C for 10 s, then 65 °C for 1 min increasing 0.5 °C per cycle until 95 °C was reached for 15 s, followed by 65 °C for 15 s. All samples were assayed in triplicate. The whole blood cytokines’ levels of gene expression were then normalized to the housekeeping gene by calculating their relative quantities to the highest CT value. For the jejunal tissue cytokine gene expression, we used the 2^−ΔΔCT^ CT method [59] to analyze the data.

**Table 7:** Primers for RT-qPCR genes based on the ovis sequences obtained from the NCBI database

Gene		Primer sequences (5′–3′)	Amplicon size	NCBI accession no.
CXCL8	F:	AGAGAGCTGAGAAGCAAGATCCA	150 bp	NM_001009401.2
	R:	CCCTACACCAGACCCACACA		
IL-1β	F:	GGCAGAAGGGAAGGGAAGA	81 bp	NM_001009465
	R:	AATACAGGGGAGGCAGTTGG		
GRα	F:	TGCCAAGGGTCTGGAGATG	132 bp	NM_001114186.1
	R:	TGAGGAACTGGATGGAGGAGA		
TLR4	F:	GACCCTTGCGTACAGGTTGTT	80 bp	NM_001135930.1
	R:	GGGATGTTGTCGGGGATTT		
TNFα	F:	ACGGCGTGGAGCTGAAA	132 bp	NM_001114186.1
	R:	CTGATGGTGTGGGTGAGGAA		
IFN-γ	F:	TGGAGGACTTCAAAAGGCTGA	183 bp	NM_001009803.1
	R:	GCAGGCAGGAGAACCATTACA		
Fas	F:	GATATTGCTTGGCTTGGCTTT	167 bp	NM_001123003.1
	R:	CCAGCATTCATCTCCCCAAC		
NFκB1	F:	AGCACCACTTATGACGGAACTACA	168 bp	XM_004009667.3
	R:	GACCCCTTCATCCTCTCCATC		
NFκB2	F:	GGAGGCCAAGGAACTGAAGA	101 bp	XM_004020143.3
	R:	TCAGGGGCAGAGAGAAGGAG		
CD62L	F:	CGGAGAAGCACGGTTGATG	198 bp	XM_012187246.2
	R:	CAAAGAGGGGACAGAAGGAGAAG		
β-actin	F:	CCTGCGGCATTCACGAA	134 bp	NM_001009784.2
	R:	GCGGATGTCGACGTCACA		

### DNA extraction, PCR amplification, and sequencing

Total genomic DNA from the samples was extracted using the Omega E.Z.N.A.™ Stoll DNA kit (Omega Bio-Tek, Norcross, GA, USA). DNA concentration and purity were monitored on 1%-agarose gels, and the final concentrations of extracted DNA were determined in a Nano-Drop 2000 spectrophotometer (Thermo Scientific, Wilmington, USA). According to its concentration, the DNA was diluted to 1 ng/μL with sterile water. 16S-rRNA genes of distinct regions were then amplified using a specific primer (341F: CCTAYGGGRBGCASCAG; 806R: GGACTACNNGGGTATCTAAT) with the barcode. All the PCR reactions were carried out using a Phusion® High-Fidelity PCR Master Mix (New England Biolabs, Essex, USA). Samples with a bright main strip between 400 and 450 bp were selected for use in further experiments. The PCR products were mixed in equidensity ratios, and these mixture PCR products purified with a Qiagen Gel Extraction Kit (Qiagen, Duesseldorf, Germany). Sequencing libraries were generated with a TruSeq® DNA PCR-Free Sample Preparation Kit (Illumina, San Diego, USA), following manufacturer’s recommendations, and index codes added. The library quality was assessed on a coupled Qubit 2.0 Fluorometer (Thermo Scientific, Wilmington, USA) and Agilent Bioanalyzer 2100 system (Agilent Technologies, Palo Alto, USA). Finally, the library was sequenced on an Illumina HiSeq 2500 platform, which generated the 250 bp paired-end reads.

### Analysis of 16S rDNA sequencing data

Raw sequences were filtered through a quality control pipeline with quality scores > 30 retained for further analyses. Quality filtering on the raw tags was performed under specific filtering conditions to obtain high-quality clean tags [60] according to the QIIME (v1.7.0, http://qiime.org/index.html) [17] quality-controlled process. These tags were then compared with the reference database (Gold database, http://drive5.com/uchime/uchime_download.html) using the UCHIME algorithm (http://www.drive5.com/usearch/manual/uchime_algo.html) [61] to detect chimera sequences: these were removed [62], to obtain the effective tags. Sequence analysis was performed in Uparse software (v7.0.1001, http://drive5.com/uparse/) [63], with those sequences with ≥97% similarity assigned to the same operational taxonomic units (OTUs). The representative sequence for each OTU was screened for further annotation. For each representative sequence, the Greengenes database (http://greengenes.lbl.gov/Download/) [64] was used based on the RDP classifier (v2.2, http://sourceforge.net/projects/rdp-classifier/) [65] algorithm to annotate the taxonomic information. The OTU abundances were normalized using a standard sequence number corresponding to the sample with the fewest sequences. Subsequent analyses of the alpha and beta diversity of the microbiota were performed basing on this output-normalized data. Alpha diversity was investigated by analyzing the species diversity of a given sample, expressed by six indices: Observed-species, Chao1, Shannon, Simpson, ACE, and coverage. All these indices were calculated with QIIME (v1.7.0) and displayed with R software (v2.15.3). Beta diversity analysis evaluated the differences among the samples in species complexity, using weightings and also calculated in QIIME. Non-metric multi-dimensional scaling (NMDS) analysis, with a conventional cut-off of < 0.2 for the stress value was obtained by using the ‘vegan’ package. All sequencing data are available at NCBI (NCBI Bioproject Accession number: PRJNA432641).

### PCA and correlation analysis

The hematology, acute phase protein, norepinephrine, cortisol and whole blood cytokines genes’ expression levels were determined by principal component analysis (PCA). This cluster analysis included the data at 0, 1, 2, 3, and 7 d, with differences expressed in colors. PCA results were displayed by the ‘ade4’ package and correlations by the ‘gplots’ package, with the ‘psych’ package used to calculate *P-*values in the R software platform (v2.15.3).

### Statistical analysis

Relative gene expression values were all log_2_-transformed before their analysis. Hematology, hormone, acute phase protein, and relative gene expression were analyzed in a 2 × 2 factorial design with repeated measures, by using the PROC MIXED procedure in SAS (v9.4, SAS Institute, Cary, USA). The subject animal was the experimental block unit, treated as a repeated measures effect, and the dependence within animal was modeled using an unstructured covariance structure. The corresponding least squares means (LS-means) and standard errors of the mean (SEM) are presented to facilitate interpretation of the results. The first sample (i.e., at − 3 d) was used as the baseline covariate in the statistical analysis. Differences between means were tested using the PDIFF option in PROC MIXED. The responses at 1, 2, 3, 7, 14, and 21 d were respectively compared with the baseline (= 0 d). Superscripts a, b, c within rows are used to indicate the LS-means differed by *P* < 0.05, *P* < 0.01, and *P* < 0.001, respectively; the x, y labels within columns indicate the LS-means differed by *P* < 0.05.The bacterial data for the C and H groups were compared using an independent-sample *t*-test implemented in SAS. Means were considered significantly different at the *P* < 0.05 level but trends also reported if 0.05 < *P* < 0.10.

## Abbreviations

cDNA: Complementary DNA
CP: Crude protein
CT: Cycle threshold
HGB: Hemoglobin
HP: Haptoglobin
HPA: Hypothalamo-pituitary-adrenal
IFN-γ: Interferon-γ
IL1: Interleukin-1
K_3_EDTA: K_3_Ethylenediaminetetraacetic acid
LH: Lithium Heparin
LPS: Lipopolysaccharides
MR: Milk replacer
N:L: Neutrophil: lymphocyte
NE: Norepinephrine
NMDS: Non-metric multi-dimensional scaling
OTUs: Operational taxonomic units
PCA: Principal component analysis
RBC: Red blood cell
SEM: Standard errors of the mean
TNFα: Tumor necrosis factor-α

## References

[1] Weary DM, Jasper J, Hotzel MJ (2008). Understanding weaning distress. Appl Anim Behav Sci.

[2] O’Loughlin A, Lynn DJ, Mcgee M, Doyle S, McCabe M, Earley B (2012). Transcriptomic analysis of the stress response to weaning at housing in bovine leukocytes using RNA-seq technology. BMC Genomics.

[3] O’Loughlin A, McGee M, Doyle S, Earley B (2014). Biomarker responses to weaning stress in beef calves. Res Vet Sci.

[4] O’Loughlin A, McGee M, Waters SM, Doyle S, Earley B (2011). Examination of the bovine leukocyte environment using immunogenetic biomarkers to assess immunocompetence following exposure to weaning stress. BMC Vet Res.

[5] Lynch EM, Earley B, McGee M, Doyle S (2010). Effect of abrupt weaning at housing on leukocyte distribution, functional activity of neutrophils, and acute phase protein response of beef calves. BMC Vet Res.

[6] Ballou MA (2012). Immune responses of Holstein and Jersey calves during the preweaning and immediate postweaned periods when fed varying planes of milk replacer. J Dairy Sci.

[7] Garrett WS, Gordon JI, Glimcher LH (2010). Homeostasis and inflammation in the intestine. Cell.

[8] Hodgson PD, Aich P, Stookey J, Popowych Y, Potter A, Babiuk L, Griebel PJ (2012). Stress significantly increases mortality following a secondary bacterial respiratory infection. Vet Res.

[9] Hickey MC, Drennan M, Earley B (2003). The effect of abrupt weaning of suckler calves on the plasma concentrations of cortisol, catecholamines, leukocytes, acute-phase proteins and in vitro interferon-gamma production. J Anim Sci.

[10] Burke NC, Scaglia G, Boland HT, Swecker WS (2009). Influence of two-stage weaning with subsequent transport on body weight, plasma lipid peroxidation, plasma selenium, and on leukocyte glutathione peroxidase and glutathione reductase activity in beef calves. Vet Immunol Immunopathol.

[11] Hulbert LE, Cobb CJ, Carroll JA, Ballou MA (2011). The effects of early weaning on innate immune responses of Holstein calves. J Dairy Sci.

[12] Johnston D, Kenny DA, Kelly AK, McCabe MS, McGee M, Waters SM, Earley B (2016). Characterisation of haematological profiles and whole blood relative gene expression levels in Holstein-Friesian and Jersey bull calves undergoing gradual weaning. Animal : an international journal of animal bioscience.

[13] Johnston D, Earley B, Cormican P, Kenny DA, McCabe MS, Kelly AK, McGee M, Waters SM (2016). Characterisation of the whole blood mRNA transcriptome in Holstein-Friesian and Jersey calves in response to gradual weaning. PLoS One.

[14] Lynch EM, McGee M, Doyle S, Earley B (2012). Effect of pre-weaning concentrate supplementation on peripheral distribution of leukocytes, functional activity of neutrophils, acute phase protein and behavioural responses of abruptly weaned and housed beef calves. BMC Vet Res.

[15] Kelley KW, Johnson RW, Dantzer R (1994). Immunology discovers physiology. Vet Immunol Immunopathol.

[16] Dhabhar FS (2009). A hassle a day may keep the pathogens away: the fight-or-flight stress response and the augmentation of immune function. Integr Comp Biol.

[17] Caporaso JG, Kuczynski J, Stombaugh J, Bittinger K, Bushman FD, Costello EK, Fierer N, Pena AG, Goodrich JK, Gordon JI (2010). QIIME allows analysis of high-throughput community sequencing data. Nat Methods.

[18] Keller M, Spanou Z, Schaerli P, Britschgi M, Yawalkar N, Seitz M, Villiger PM, Pichler WJ (2005). T cell-regulated neutrophilic inflammation in autoinflammatory diseases. J Immunol.

[19] Johnson JD, Campisi J, Sharkey CM, Kennedy SL, Nickerson M, Greenwood BN, Fleshner M (2005). Catecholamines mediate stress-induced increases in peripheral and central inflammatory cytokines. Neuroscience.

[20] Turnbaugh PJ, Hamady M, Yatsunenko T, Cantarel BL, Duncan A, Ley RE, Sogin ML, Jones WJ, Roe BA, Affourtit JP (2009). A core gut microbiome in obese and lean twins. Nature.

[21] Houghteling PD, Walker WA (2015). Why is initial bacterial colonization of the intestine important to Infants’ and Children’s health?. J Pediatr Gastr Nutr.

[22] Xu J, Lian FM, Zhao LH, Zhao YF, Chen XY, Zhang X, Guo Y, Zhang CH, Zhou Q, Xue ZS (2015). Structural modulation of gut microbiota during alleviation of type 2 diabetes with a Chinese herbal formula. Isme J.

[23] Wang TT, Cai GX, Qiu YP, Fei N, Zhang MH, Pang XY, Jia W, Cai SJ, Zhao LP (2012). Structural segregation of gut microbiota between colorectal cancer patients and healthy volunteers. Isme J..

[24] Turnbaugh PJ, Ley RE, Hamady M, Fraser-Liggett CM, Knight R, Gordon JI (2007). The human microbiome project: exploring the microbial part of ourselves in a changing world. Nature.

[25] Sovran B, Lu P, Loonen LM, Hugenholtz F, Belzer C, Stolte EH, Boekschoten MV, van Baarlen P, Smidt H, Kleerebezem M (2016). Identification of commensal species positively correlated with early stress responses to a compromised mucus barrier. Inflamm Bowel Dis.

[26] Malmuthuge N, Griebel PJ, Guan le L (2015). The gut microbiome and its potential role in the development and function of newborn calf gastrointestinal tract. Front Vet Sci.

[27] Fischer AJ, Song Y, He Z, Haines DM, Guan LL, Steele MA (2018). Effect of delaying colostrum feeding on passive transfer and intestinal bacterial colonization in neonatal male Holstein calves. J Dairy Sci.

[28] Budzynska M, Weary DM (2008). Weaning distress in dairy calves: effects of alternative weaning procedures. Appl Anim Behav Sci.

[29] Eckert E, Brown HE, Leslie KE, DeVries TJ, Steele MA (2015). Weaning age affects growth, feed intake, gastrointestinal development, and behavior in Holstein calves fed an elevated plane of nutrition during the preweaning stage. J Dairy Sci.

[30] Terré M, Devant M, Bach A (2007). Effect of level of milk replacer fed to Holstein calves on performance during the preweaning period and starter digestibility at weaning. Livest Sci.

[31] Southwick SM, Bremner JD, Rasmusson A, Morgan CA, Arnsten A, Charney DS (1999). Role of norepinephrine in the pathophysiology and treatment of posttraumatic stress disorder. Biol Psychiatry.

[32] Angen O, Thomsen J, Larsen LE, Larsen J, Kokotovic B, Heegaard PMH, Enemark JMD (2009). Respiratory disease in calves: microbiological investigations on trans-tracheally aspirated bronchoalveolar fluid and acute phase protein response. Vet Microbiol.

[33] Murata H, Shimada N, Yoshioka M (2004). Current research on acute phase proteins in veterinary diagnosis: an overview. Vet J.

[34] Carroll JA, Arthington JD, Chase CC (2009). Early weaning alters the acute-phase reaction to an endotoxin challenge in beef calves. J Anim Sci.

[35] Carroll JA, Forsberg NE (2007). Influence of stress and nutrition on cattle immunity. The veterinary clinics of North America food animal. Practice.

[36] Zecca M, Nobili B, Ramenghi U, Perrotta S, Amendola G, Rosito P, Jankovic M, Pierani P, De Stefano P, Bonora MR (2003). Rituximab for the treatment of refractory autoimmune hemolytic anemia in children. Blood.

[37] Kim MH, Yang JY, Upadhaya SD, Lee HJ, Yun CH, Ha JK (2011). The stress of weaning influences serum levels of acute-phase proteins, iron-binding proteins, inflammatory cytokines, cortisol, and leukocyte subsets in Holstein calves. J Vet Sci.

[38] Weber PS, Toelboell T, Chang LC, Tirrell JD, Saama PM, Smith GW, Burton JL (2004). Mechanisms of glucocorticoid-induced down-regulation of neutrophil L-selectin in cattle: evidence for effects at the gene-expression level and primarily on blood neutrophils. J Leukoc Biol.

[39] Lynch EM, Earley B, McGee M, Doyle S (2010). Characterisation of physiological and immunological responses in beef cows to abrupt weaning and subsequent housing. BMC Vet Res.

[40] Lichtman AH, Chin J, Schmidt JA, Abbas AK (1988). Role of Interleukin-1 in the activation of lymphocytes-T. Proc Natl Acad Sci U S A.

[41] Bemelmans MHA, vanTits LJH, Buurman WA (1996). Tumor necrosis factor: function, release and clearance. Crit Rev Immunol.

[42] Boehm U, Klamp T, Groot M, Howard JC (1997). Cellular responses to interferon-gamma. Annu Rev Immunol.

[43] Schroder K, Hertzog PJ, Ravasi T, Hume DA (2004). Interferon-gamma: an overview of signals, mechanisms and functions. J Leukoc Biol.

[44] Carta S, Semino C, Sitia R, Rubartelli A (2017). Dysregulated IL-1beta secretion in autoinflammatory diseases: a matter of stress?. Front Immunol.

[45] Elenkov IJ, Iezzoni DG, Daly A, Harris AG, Chrousos GP (2005). Cytokine dysregulation, inflammation and well-being. Neuroimmunomodulation.

[46] Sporer KRB, Burton JL, Earley B, Crowe MA (2007). Transportation stress in young bulls alters expression of neutrophil genes important for the regulation of apoptosis, tissue remodeling, margination, and anti-bacterial function. Vet Immunol Immunopathol.

[47] Seki E, De Minicis S, Osterreicher CH, Kluwe J, Osawa Y, Brenner DA, Schwabe RF (2007). TLR4 enhances TGF-beta signaling and hepatic fibrosis. Nat Med.

[48] Trichet VV (2010). Nutrition and immunity: an update. Aquac Res.

[49] Le Chatelier E, Nielsen T, Qin J, Prifti E, Hildebrand F, Falony G, Almeida M, Arumugam M, Batto JM, Kennedy S (2013). Richness of human gut microbiome correlates with metabolic markers. Nature.

[50] Nylund L, Satokari R, Nikkila J, Rajilic-Stojanovic M, Kalliomaki M, Isolauri E, Salminen S, de Vos WM (2013). Microarray analysis reveals marked intestinal microbiota aberrancy in infants having eczema compared to healthy children in at-risk for atopic disease. BMC Microbiol.

[51] Maynard CL, Elson CO, Hatton RD, Weaver CT (2012). Reciprocal interactions of the intestinal microbiota and immune system. Nature.

[52] Gophna U, Sommerfeld K, Gophna S, Doolittle WF, Veldhuyzen van Zanten SJ (2006). Differences between tissue-associated intestinal microfloras of patients with Crohn's disease and ulcerative colitis. J Clin Microbiol.

[53] Manichanh C, Rigottier-Gois L, Bonnaud E, Gloux K, Pelletier E, Frangeul L, Nalin R, Jarrin C, Chardon P, Marteau P (2006). Reduced diversity of faecal microbiota in Crohn's disease revealed by a metagenomic approach. Gut.

[54] Escobar JC, Bhavnani D, Trueba G, Ponce K, Cevallos W, Eisenberg J (2012). Plesiomonas shigelloides infection, Ecuador, 2004-2008. Emerg Infect Dis.

[55] Bodhidatta L, McDaniel P, Sornsakrin S, Srijan A, Serichantalergs O, Mason CJ (2010). Case-control study of diarrheal disease etiology in a remote rural area in Western Thailand. Am J Trop Med Hyg.

[56] Yue XX, Diao QY, Ma CH, Deng KD, Tu Y, Jiang CG, Du HF (2011). Effects of feeding levels of a milk replacer on digestion and metabolism of nutrients, and serum biochemical indexes in lambs. Sci Agric Sin.

[57] Bustin SA, Beaulieu JF, Huggett J, Jaggi R, Kibenge FS, Olsvik PA, Penning LC, Toegel S (2010). MIQE precis: practical implementation of minimum standard guidelines for fluorescence-based quantitative real-time PCR experiments. BMC Mol Biol.

[58] Pfaffl MW (2001). A new mathematical model for relative quantification in real-time RT-PCR. Nucleic Acids Res.

[59] Livak KJ, Schmittgen TD (2001). Analysis of relative gene expression data using real-time quantitative PCR and the 2(T)(−Delta Delta C) method. Methods.

[60] Bokulich NA, Subramanian S, Faith JJ, Gevers D, Gordon JI, Knight R, Mills DA, Caporaso JG (2013). Quality-filtering vastly improves diversity estimates from Illumina amplicon sequencing. Nat Methods.

[61] Edgar RC, Haas BJ, Clemente JC, Quince C, Knight R (2011). UCHIME improves sensitivity and speed of chimera detection. Bioinformatics.

[62] Haas BJ, Gevers D, Earl AM, Feldgarden M, Ward DV, Giannoukos G, Ciulla D, Tabbaa D, Highlander SK, Sodergren E (2011). Chimeric 16S rRNA sequence formation and detection in sanger and 454-pyrosequenced PCR amplicons. Genome Res.

[63] Edgar RC (2013). UPARSE: highly accurate OTU sequences from microbial amplicon reads. Nat Methods.

[64] DeSantis TZ, Hugenholtz P, Larsen N, Rojas M, Brodie EL, Keller K, Huber T, Dalevi D, Hu P, Andersen GL (2006). Greengenes, a chimera-checked 16S rRNA gene database and workbench compatible with ARB. Appl Environ Microbiol.

[65] Wang Q, Garrity GM, Tiedje JM, Cole JR (2007). Naive Bayesian classifier for rapid assignment of rRNA sequences into the new bacterial taxonomy. Appl Environ Microbiol.

